# Fabrication of Layered SiC/C/Si/MeSi_2_/Me Ceramic–Metal Composites via Liquid Silicon Infiltration of Metal–Carbon Matrices

**DOI:** 10.3390/ma17030650

**Published:** 2024-01-29

**Authors:** Alexei Kaledin, Sergey Shikunov, Julia Zubareva, Ivan Shmytko, Boris Straumal, Vladimir Kurlov

**Affiliations:** Osipyan Institute of Solid State Physics of the Russian Academy of Sciences, Ac. Osipyan Str. 2, 142432 Chernogolovka, Russia; kaledin@issp.ac.ru (A.K.); shikunov@issp.ac.ru (S.S.); zubarevajn@issp.ac.ru (J.Z.); kurlov@issp.ac.ru (V.K.)

**Keywords:** layered composites, metal ceramic composition, refractory silicides, silicon carbide ceramics, liquid silicon infiltration, microstructure, flexural strength, X-ray phase analysis

## Abstract

The growing demand for composite materials capable of enduring prolonged loads in high-temperature and aggressive environments presents pressing challenges for materials scientists. Ceramic materials composed of silicon carbide largely possess high mechanical strength at a relatively low density, even at elevated temperatures. However, they are inherently brittle in nature, leading to concerns about their ability to fracture. The primary objective of this study was to develop a novel technique for fabricating layered composite materials by incorporating SiC-based ceramics, refractory metals, and their silicides as integral constituents. These layered composites were produced through the liquid-phase siliconization method applied to metal–carbon blanks. Analysis of the microstructure of the resultant materials revealed that when a metal element interacts with molten silicon, it leads to the formation of a layer of metal silicide on the metal’s surface. Furthermore, three-point bending tests exhibited an enhancement in the bending strength of the layered composite in comparison to the base silicon carbide ceramics. Additionally, the samples demonstrated a quasi-plastic nature during the process of destruction.

## 1. Introduction

The growing demand for composite materials capable of enduring prolonged loads in high-temperature and aggressive environments presents pressing challenges for materials scientists. These challenges necessitate advancements not only in the inherent properties of materials but also in the methodologies employed for their production. Crucial requisites include elevated strength characteristics, crack resistance, and heat resistance. Silicon carbide ceramics are extensively employed due to their unique combination of high strength, physicochemical attributes, tribological robustness, and thermophysical properties. These ceramics find widespread use in various applications, such as face seals in pumps, heat exchange equipment, components of gas turbine engines (GTEs), fusion reactors, high-temperature equipment, catalyst supports, heaters, burners, thermocouple covers, nozzles, etc. [1,2,3,4,5,6,7,8,9]. The production of silicon carbide ceramics involves using primary silicon carbide powders or carbon blanks. Ceramic SiC material is produced via hot pressing [10,11,12] at temperatures exceeding 2000 °C. This method ensures the creation of high-density materials with superior strength. It enables their operation at temperatures up to 1500 °C in an oxidizing atmosphere and up to 1700 °C in an inert environment. However, the mechanical processing of high-temperature-sintered workpieces is labor-intensive and necessitates the use of expensive diamond tools. Gas-phase siliconization of pre-formed porous workpieces offers a feasible way to attain ceramic composite material of intricate geometries without the need for finishing mechanical processes. Nonetheless, this process is costly, time-consuming, and frequently results in the final material containing a considerable proportion of enclosed pores [13,14,15]. One of the most technologically straightforward methods for manufacturing silicon carbide ceramics involves liquid-phase sintering using specific additives [16]. This process or siliconization with molten silicon employs porous workpieces pre-formed from primary silicon carbide powders, a blend of carbon powders, or their combinations [17,18,19,20,21,22].

Ceramic materials composed of silicon carbide largely fulfill several requirements. They boast high mechanical strength at a relatively low density, even at elevated temperatures. However, they are inherently brittle in nature, leading to concerns about their propensity to fracture. Ceramic materials are prone to sudden and catastrophic failure under an applied load due to the low fracture toughness [23]. All engineered structural materials require both high strength and high toughness, but these properties are usually mutually exclusive. To improve the toughness and reduce the sensitivity to defects in ceramic materials, the challenge of enhancing the crack resistance of both oxide and carbide ceramics is tackled through the development of composite materials based on these ceramics. Continuously reinforced composite materials, characterized by an anisotropic or orthotropic structure, exhibit the highest levels of crack resistance.

Certainly, enhancing impact toughness and crack resistance can be effectively achieved by utilizing composites featuring a SiC ceramic matrix reinforced with various continuous fibers. The selection of these reinforcing fibers relies on their intended application. For instance, ceramic composites reinforced with carbon fiber (Cf/SiC) are suitable for short-term uses, such as thermal protection systems in aerospace vehicles, aircraft braking systems, components in gas turbine engines, among others [4,24]. Alternatively, the silicon carbide fiber-reinforced silicon carbide matrix (SiC/SiC) composite system has been specifically developed for prolonged operation at high temperatures. This category of materials exhibits impressive crack resistance and maintains structural integrity up to 1400 °C [25,26]. SiC/SiC composites find effective application in gas turbine engines, jet engines, and as integral components in high-temperature gas-cooled reactors [4,27,28].

Among composite materials, layered ceramics are particularly noteworthy [29,30]. Layered structure is an emerging and effective method, which is different from the conventional way to significantly improve the fracture toughness of ceramic materials. Also, layered ceramics can improve toughness and enhance the energy dissipation even further by guiding and deflecting the cracks [31]. The strengthening mechanism of carbide-, boride- nitride-, and oxide-based layered materials [32,33,34,35,36,37,38,39] with various structural designs can be divided into two categories, depending on the strength of the interfacial bond: for layered ceramics with strong interfaces, cracks are mainly deflected due to residual stresses in the compressive layers [33,35,40,41]; for layered ceramics with weak interfaces, delamination cracks and crack deflection are the source of energy dissipation during fracture, and interfacial fracture resistance is introduced to predict delamination and crack propagation [31,42].

Layered composite materials are sophisticated structures, crafted by combining two or more materials with contrasting properties, bonded together to create a singular structure with enhanced performance characteristics. This advanced engineering technique involves stacking various layers of dissimilar materials, such as metals, ceramics, polymers, or fibers, to exploit the strengths of each constituent material and produce a composite with tailored properties well suited for specific applications. The construction of layered composites involves a meticulous process aimed at achieving a synergistic blend of properties. Each layer contributes distinct attributes to the overall structure. For instance, fibers like carbon or glass provide excellent tensile strength and stiffness, while polymers offer flexibility and impact resistance. Metals such as aluminum or titanium impart toughness and durability, while ceramics like silicon carbide contribute high-temperature resistance and hardness. One of the primary advantages of layered composites is their ability to combine diverse properties, thereby offering a unique combination of strength, durability, lightweight nature, and other desirable characteristics.

Layered composite materials represent a highly promising avenue for applications requiring substantial stress intensity factor values (around 20 MPa × m^1/2^) [38,43]. The amalgamation of ceramics with refractory metals, possessing ductility across a wide temperature range, into a composite layered material like SiC holds considerable potential in establishing a new class of materials, exhibiting quasi-plastic fracture behavior and enhanced crack resistance compared to ceramics. Among the array of refractory metals, tungsten, molybdenum, and niobium stand out as the most accessible and commonly used metals in structural configurations. Additionally, owing to its high effective strength (yield strength-to-density ratio), titanium and its derivatives are frequently employed both as structural elements and as constituents in related composite materials. Notably, the coefficients of thermal expansion (CTE) for these metals approximate the value of SiC and fall within the same order of magnitude (4.5 × 10^−6^/K for SiC, 4.4 × 10^−6^/K for W, 5.09 × 10^−6^/K for Mo, 7.2 × 10^−6^/K for Nb, and 8.4 × 10^−6^/K for Ti) [44]. Consequently, the most suitable combinations emerge with Mo–SiC and W–SiC, where the difference in CTE remains minimal. Nonetheless, the high density of tungsten significantly constrains the applicability of the composite materials incorporating it.

This paper introduces a novel technique for fabricating layered composite materials by incorporating SiC-based ceramics, refractory metals, and their silicides as integral constituents. The primary objective of this study was twofold: first, to showcase the inherent feasibility of producing this material class via the liquid silicon infiltration of metal–carbon blanks, and, second, to validate the concept of enhancing the crack resistance of SiC ceramics by integrating them with malleable metal components.

## 2. Experimental

### 2.1. Composite Fabrication

Samples of layered composite materials were manufactured to investigate their microstructure, phase composition, and mechanical strength through a process involving the liquid-phase siliconization of workpieces. These blanks possess a layered architecture and comprise a blend of refractory metal foils and porous carbon. The metal–carbon blanks were derived by pressing metal foils alongside a mixture of fine carbon powders and a polymer binder. This methodology draws from the technique employed in producing ceramic SiC materials and their components [45,46], which facilitated overcoming challenges associated with manufacturing complex-shaped products using SiC ceramics. The ceramic material is obtained through a non-shrink siliconization process (involving the impregnation of a carbon-containing workpiece with molten silicon to yield silicon carbide) of porous carbon materials. This method enables the shaping of the final product during the carbon blank phase, minimizing the need for extensive finishing of ceramic parts and substantially reducing production costs. Specifically, specialized porous graphite can serve as the initial carbon blank, formulated by blending and compressing carbon powders with an organic coking binder.

The utilization of this method allows for extensive control over the density and pore structure of the carbon workpiece across a broad spectrum. This control is achieved by modifying the pressing pressure, varying graphite powder fractions, and adjusting the quantity of binder used during the production of synthetic porous graphite. Consequently, this flexibility enables an alteration in the phase composition (SiC/C/Si phase ratio) and the structure of the ceramic within a wide range, tailored to meet specific requirements dictated by the operational conditions of a given product. Following the siliconization process of the carbon matrices, SiC ceramic-based materials were obtained with densities ranging from 2.32 g/cm^3^ for the densest graphite workpiece (initial density of 1.46 g/cm^3^) to 3.1 g/cm^3^ for a carbon workpiece with an initial density of 0.9 g/cm^3^.

**Figure 1:**
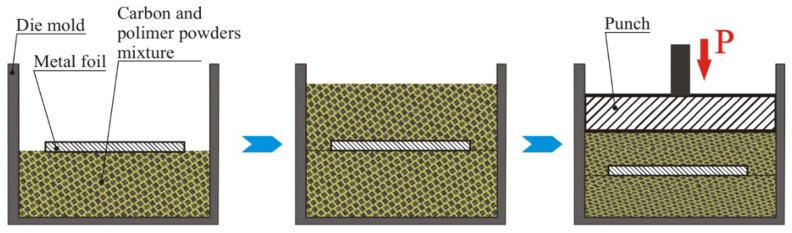
Scheme for obtaining carbon porous blanks reinforced with metal foil.

As the foundation for producing layered composites, a material with 50–55 vol.% SiC content and a relatively high residual carbon content of 30–35 vol.% was selected. This composition was chosen for its high resistance to thermal shock. In the manufacturing process, foils of Mo and Nb measuring 0.5 mm in thickness, as well as Ti foils measuring 0.4 mm thick, were utilized as the metallic component in the composite materials. These foils were arranged within molds, interspersed with layers of carbon charge comprising graphite powders in the 100–200 µm fraction range, along with a polymer binder, as illustrated in Figure 1. Subsequently, the resultant mixture underwent compression with following heat treatment to polymerize the binder.

Subsequently, this process involved high-temperature treatment, during which the polymer binder underwent partial coking, leading to the development of a porous structure throughout the carbon component of the workpieces. Following this phase, the porous metal–carbon blanks underwent liquid-phase siliconization. This operation occurred within an inert environment at a temperature of 1500 °C, allowing the silicon melt to interact with both the carbon and metal elements, resulting in the creation of silicon carbide and metal silicides. Consequently, the outcome was the production of metal–ceramic blanks, showcasing a layered structure, comprising alternating layers of silicon carbide ceramics, refractory metals, and their respective silicides, which are close in terms of elastic modulus to that of SiC. Among the silicides, the molybdenum-, titanium-, and niobium-based multiphase alloys and composites were found to be the most promising [47]. They have high melting points, ability of strength retention, along with impressive oxidation resistance at elevated temperatures, which are considered desirable.

### 2.2. Characterization

The phases and microstructural features of the polished samples were evaluated on a Zeiss Supra 50 VP high-resolution scanning electron microscope (Carl Zeiss, Kelsterbach, Germany) combined with an INCA Energy+ microanalysis system. The analysis of the phase composition was carried out using both a chemical composition analyzer built into the SEM and a Dron-3M X-ray diffractometer (Burevestnik Ltd., S.-Petersburg, Russia) with Cu-Kα radiation. The structural state of the samples was also recorded on a D500 X-ray diffractometer (Siemens, Erlangen, Germany) using the Bragg–Brentano scheme. Cu-Kα radiation monochromatized by output graphite monochromator (Siemens, Erlangen, Germany) was used. Diffraction pattern visualization was produced with help of powder diffraction software Match, version 1.11 k, which was developed by Dr. Holger Putz and Dr. Klaus Brandenburg from University of Bonn, Germany. Phase identification was fulfilled with help of the PDF-2 (Powder Diffraction Files, version 2.0) and the COD (version 3.1) databases (ICDD, Newtown, PA, USA).

The flexural strength of the materials was evaluated at room temperature in a 3-point bending test using specimens 3 × 5 × 35 mm^3^ in size with a support span of 25 mm. The mechanical tests were carried out on an electromechanical testing machine (universal testing machine I1147 M, 50 kN, Tochpribor, Ivanovo, Russia) with a force sensor of 50 kN. To evaluate the impact of the metal component on the physical and mechanical properties of silicon carbide ceramics, tests were conducted on SiC-based ceramics derived from graphite powders of 100–200 µm fractions and a polymer binder, forming the ceramic foundation of the developed composites.

## 3. Results and Discussion

### 3.1. Phase Composition and Microstructure

**Figure 2:**
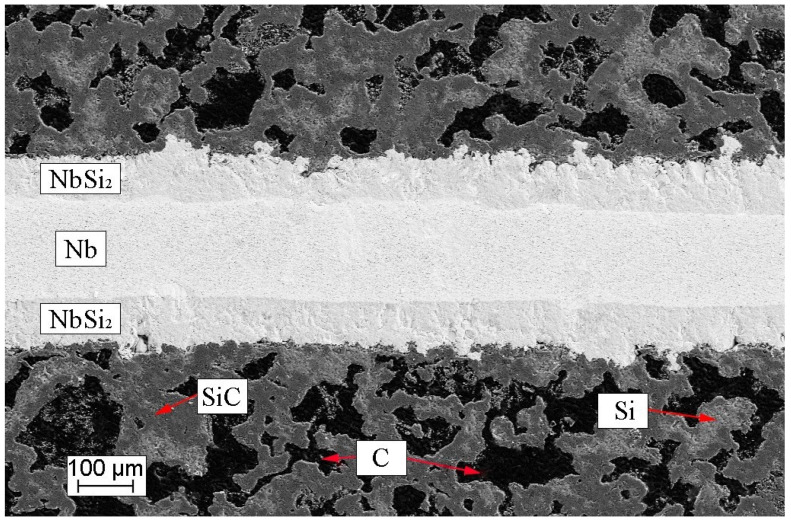
Microstructure of SiC-based ceramic with Nb/NbSi_2_ layers.

The analysis of the microstructure in the obtained composite material samples revealed the development of a layered structure due to the interaction between the refractory metal foils and molten silicon during the impregnation process. This resulted in the formation of a structured composition, SiC/MeSi_2_/Me, where ‘Me’ denotes the original metal (Mo, Nb, Ti). At the core of the sample cross-section, there is a layer of refractory metal, surrounded by layers of its respective silicide. These silicide layers are intricately bonded to layers of SiC ceramics. The ceramic SiC matrix is a composite resembling siliconized graphite, comprising 50–55 vol.% SiC, 30–35 vol.% carbon (crystalline carbon in the form of a residual graphite and amorphous carbon constituting a carbonized polymer binder) and 10–15 vol.% silicon (Figure 2).

**Figure 3:**
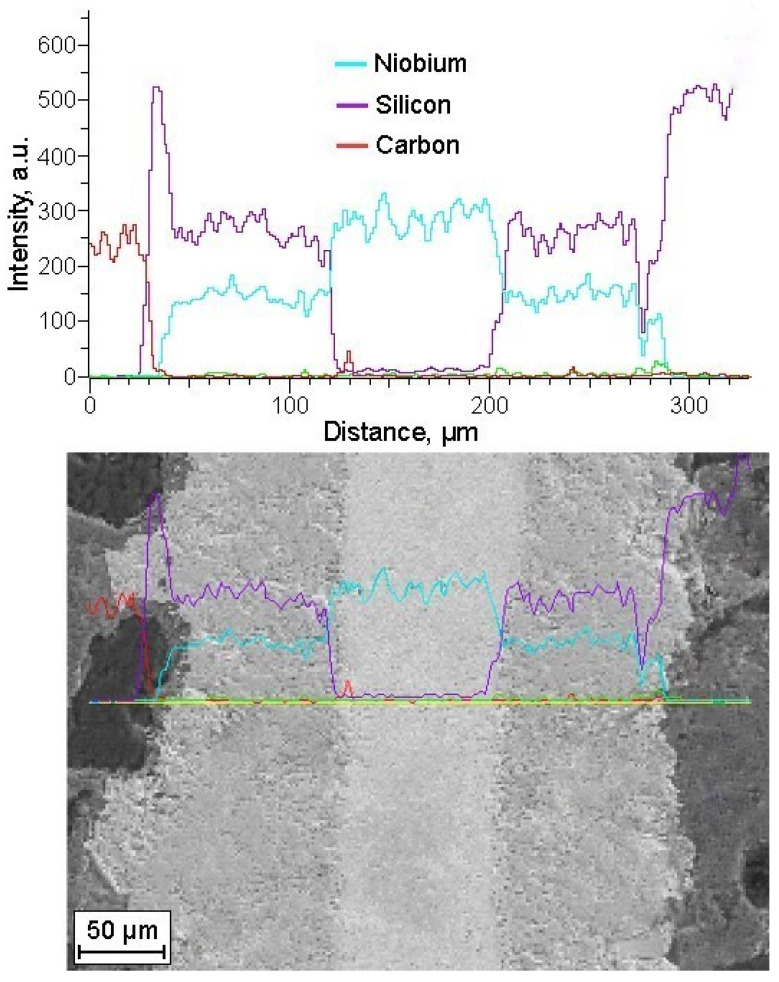
Elemental analysis of layered SiC/C/Si/Nb/NbSi_2_ composite. Yellow straight line in the micrograph shows the position of analysis, the green, turquoise, red and green curves repeat the concentration curves for Nb, Si, C and Mo from the upper part of the figure.

The elemental analysis of the intermediary silicide layers indicated an atomic ratio of metal to silicon approximately close to 1:2, aligning with the stoichiometric proportion of refractory metal disilicides (MoSi_2_, NbSi_2_, TiSi_2_), as depicted in Figure 3. The disilicide layer’s thickness varies within a range of 75–200 µm. At the same time, these layers provide good bonding with both the metal and the ceramic matrix. In this case, the construction of a toughness-enhanced layered system established a control base, not only through a weak interface to promote crack deflection but also via a chemically compatible interface to avoid the buildup of internal stresses.

**Figure 4:**
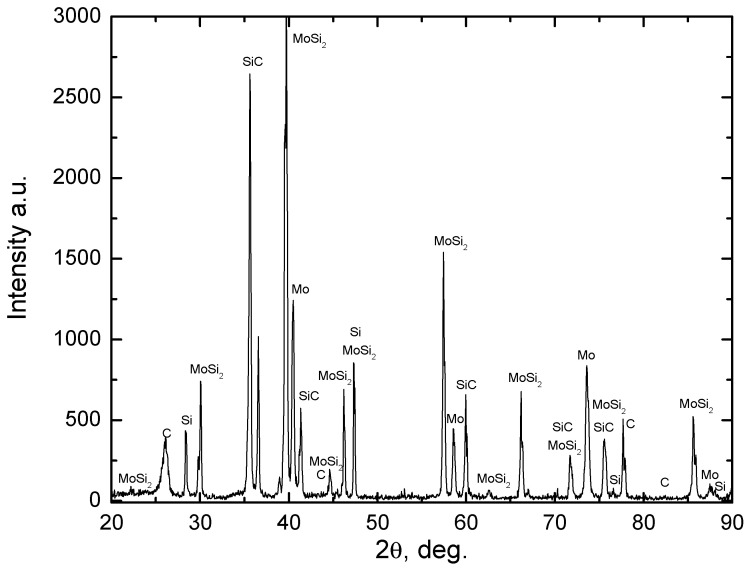
XRD pattern of the SiC-based ceramic with Mo/MoSi_2_ layers.

For a more precise assessment of the phase composition within the silicide layer and its adjacent regions, an X-ray analysis of their cross-section was conducted for all variants of the developed materials—Mo/MoSi_2_/SiC, Nb/NbSi_2_/SiC, and Ti/TiSi_2_/SiC. Figure 4 shows the X-ray diffraction pattern of a Mo/MoSi_2_/SiC composite sample. The observed peaks correspond to SiC phases of two polytypes, molybdenum disilicide MoSi_2_, along with residual molybdenum, free silicon, and carbon present in the ceramic component. Notably, there were no reflections identified indicative of carbides of the refractory metals used in any of the samples.

### 3.2. Flexural Strength Testing of Composite Material Samples

**Figure 5:**
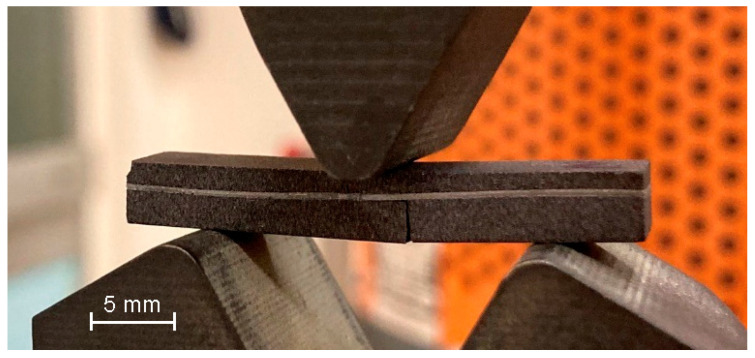
Three-point bending test conducted on a layered composite material sample.

Initially, pure samples of SiC ceramics were tested, devoid of incorporated metal foils, which, as previously indicated, comprise SiC, free silicon, and carbon in a ratio of SiC/C/Si-55/30/15 vol.%. The test outcomes of the fundamental SiC ceramics were juxtaposed with the results obtained from the metal–ceramic composite materials derived from them. During the three-point bending tests conducted on composite samples, the ceramic component failed upon reaching a critical stress level (Figure 5).

**Figure 6:**
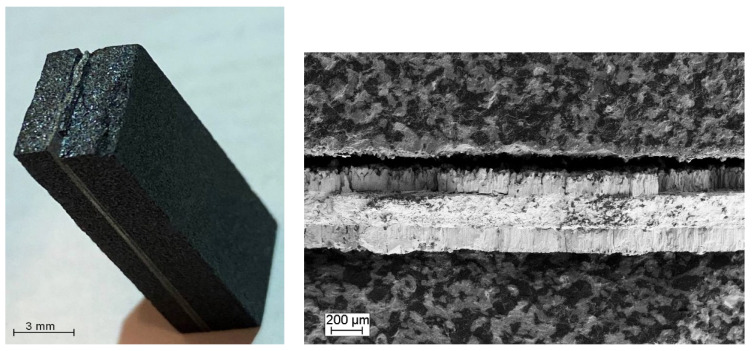
Micrographs of failure surfaces of SiC/C/Si/Nb/NbSi_2_ layered composite after testing.

Nevertheless, the metal layer functioned as a barrier for the progressing crack. Figure 6 depicts the fracture of a deteriorated layered composite sample. Subsequent plastic deformation of the metal component played a role in maintaining the load-bearing capacity of the sample, contributing to the overall quasi-plastic nature of the materials developed. The polycrystalline silicides exhibit brittle behavior at ambient temperature. However, additional slip systems become operative or dislocation climb becomes possible with an increase in temperature, which, in turn, contributes to brittle-to-ductile transition in these materials [47].

For such layered ceramics with weak interfacial bonding strength, the crack propagates due to delamination and deflection. Delamination cracks and crack deflection are the source of energy dissipation during the fracture of laminated ceramics with weak interfaces. The mechanism of toughening in layered ceramics with weak interfacial bonding strength was reported by Kovar et al. [42].

**Figure 7:**
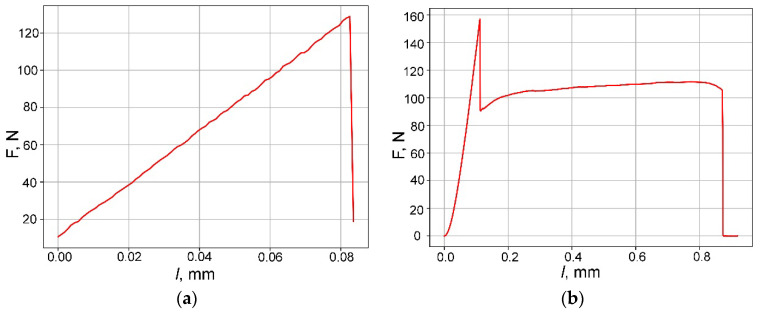
Loading diagrams at room temperature: SiC/C/Si (**a**), SiC/C/Si/Nb/NbSi_2_ (**b**) samples. The peak corresponds to the moment of destruction of the composite ceramic matrix.

A comparison of the fracture curves of samples of pure SiC ceramics and a layered composite material based on it is presented in Figure 7.

The loading diagrams at room temperature for SiC/C/Si/Mo/MoSi_2_ and SiC/C/Si/Ti/TiSi_2_ layered composites are similar to the fracture mode of loading diagrams for SiC/C/Si/Nb/NbSi_2_ and differ only in the load value. The average critical load values for each material type, as well as the failure load value of pure SiC ceramics (first raw), are listed in Table 1. It is noticeable that the flexural strength of the base ceramics not only remains unaltered but also experiences a significant increase upon the incorporation of metal-reinforcing elements into its structure.

Additionally, the developed method for fabricating layered composite materials offers the flexibility to adjust both the thickness and the quantity of metal layers, consequently altering the cross-sectional configuration of the samples. When utilizing foils with a thickness below 200 µm, they undergo complete transformation into silicide, enabling the creation of a layered ceramic composite material exhibiting a quasi-plastic fracture behavior at elevated temperatures (beyond the viscoelastic transition temperatures of refractory metal silicides) [48,49].

**Figure 8:**
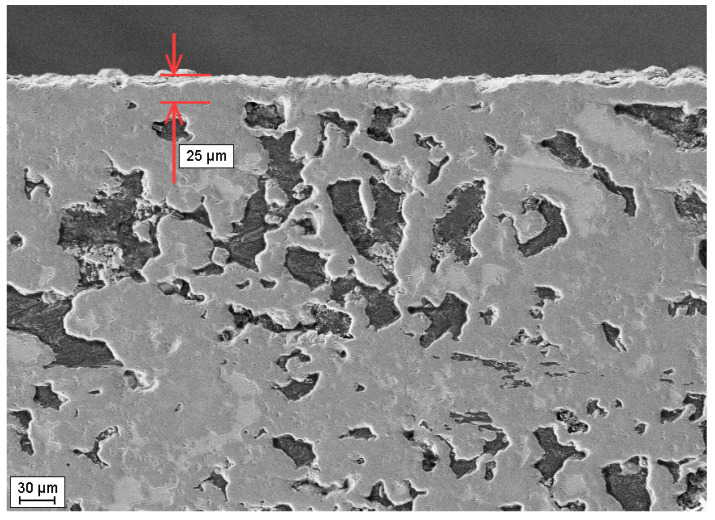
Microstructure of composite material SiC-C-Si/25/55/20 vol.% with a gas-tight SiC coating of 25 µm in thickness.

To withstand high temperatures (up to 1500 °C) in an oxidizing atmosphere, composite materials featuring a SiC/C/Si matrix, reinforced with layers of refractory metals and their silicides, are equipped with a protective gas-tight SiC coating, as illustrated in Figure 8.

The procedure for applying a gas-tight silicon carbide coating to a substrate material relies on the direct interaction between the carbon generated during the high-temperature pyrolytic decomposition of hydrocarbon molecules (such as methane) and the silicon melt present in the surface layer of the material to be coated, along with silicon vapor. The source of silicon vapor originates from the silicon melt located in the thermal zone of the furnace [50,51]. This coating serves to prevent the high-temperature oxidation of both carbon particles and residual silicon present in the material’s structure, as well as the low-temperature oxidation of metal silicides. This stability is crucial for the extended operation of such a material within a temperature range of 600–900 °C [52,53].

The thermal expansion of materials, as part of their physical properties, has a significant impact on analyzing the stress–strain state of thermally loaded structural elements and components, especially when they are made of composite materials with heterogeneous components, as is the case with multi-layered structures. The layered structure presented in this article consists of SiC-based ceramics, refractory metals Mo, Nb, Ti, and their silicides. The aforementioned difference in the coefficient of thermal expansion of these components can influence the properties of the structure when heated to high temperatures. Thermal deformations caused by differences in CTE will create internal stresses in each of the specified layers of the multilayer structure. For example, SiC, with the lowest CTE, will experience the least thermal deformation. At the same time, as the material with the highest compressive strength, SiC will be less stressed. With uniform heating of the entire multilayer system to a common temperature level, other components, namely the layers of metal and its disilicide, will also undergo temperature-induced deformation to a greater extent due to their higher CTE values compared to SiC. Internal stresses arising in the layers of disilicide due to their volumetric deformation theoretically may lead to significant deformation, up to partial layer failure, resulting in the degradation of the mechanical properties of the entire structure. Special attention should be given to the issue of cyclic thermal loads. Even slight differences in the coefficients of thermal expansion (CTE) can lead to the accumulation of stresses and, consequently, to fatigue failure. The lifespan of products made from such a material can be significantly reduced.

## 4. Conclusions

A layered composite material was developed, preserving the merits of SiC ceramics while boasting enhanced crack resistance attributed to the reinforcing metal elements capable of withstanding tension and undergoing plastic deformation. Furthermore, refractory metals, like Ti, Mo, Nb, and their silicides, can augment the composite’s heat resistance.

The concept of creating layered metal–ceramic composite materials via liquid-phase siliconization of porous carbon blanks containing a metal component was successfully demonstrated. Refractory metals, such as Mo, Nb, and Ti foils, were employed as the metallic element. The composite’s structure comprises alternating layers of SiC ceramics, a refractory metal, and its corresponding disilicide, substantiated by elemental and X-ray phase analysis. These disilicide layers, formed during the liquid-phase siliconization process on the metal surfaces, act as the bonding interface between the ceramic and metal constituents. Furthermore, both elemental and X-ray phase analyses confirmed the absence of metal carbide phases in the materials utilized.

SiC ceramics are generated during the siliconization process by the molten silicon’s interaction with the porous carbon section of the workpiece. The resulting material’s SiC content can be adjusted by altering the particle size of the carbon raw material, thereby decreasing or increasing the proportions of residual silicon and carbon. The interfacial disilicide layer thickness ranges between 100 and 200 µm, and the quantity and dimensions of the metal layers can be modified to suit the material’s specifications.

Physical and mechanical testing was conducted on the developed composite materials, specifically examining flexural strength. The baseline material selected was SiC ceramics with a SiC/C/Si ratio of 55/30/15 vol.%. An evident enhancement in flexural strength was observed in the layered composites compared to the SiC/C/Si ceramics alone (106.5 MPa for SiC/C/Si versus 149.16 MPa for the SiC/C/Si/Mo/MoSi_2_ composite material). Throughout testing, samples of the layered composite materials displayed a quasi-plastic fracture nature, affirming the potential effectiveness of the proposed concept in enhancing the crack resistance of SiC ceramics.

Layered composite materials, exemplified by the metal–ceramic SiC/C/Si/Mo/MoSi_2_, SiC/C/Si/Nb/NbSi_2_, and SiC/C/Si/Ti/TiSi_2_ composites featured in this study, hold promise in high-tech industries requiring high strength, resilience to elevated temperatures, and dynamic loads. The amalgamation of high-strength ceramics with pliable metallic components bestows upon the composite material a quasi-plastic nature during destruction, rendering it less susceptible to fracturing and enhancing its resistance against external loads, including impact-induced stress, by absorbing deformation energy through elastic metal layers. These developed layered metal–ceramic materials can serve as an alternative to the presently utilized SiC matrix composites reinforced with C and SiC fibers, especially in applications where composite weight is not a critical factor. Potential applications may include components within emerging fusion reactor casings or stationary gas turbine elements.

An imperative aspect for further research on the presented materials involves evaluating the impact of differing coefficients of thermal expansion (CTE) among all constituents—the ceramic SiC, the metal, and the interfacing layer of metal disilicide—on the material’s long-term durability. For instance, the thermal expansion coefficients of Nb and Mo disilicides fall within a similar range as those of SiC and pure metals, approximately 8.1 × 10^−6^/K and 11.7 × 10^−6^/K, respectively [54,55]. It appears pertinent to evaluate the effect of these differences in thermal expansion coefficients on the interlayer strength and flexural properties of materials subjected to elevated temperatures and cyclic thermal loads, considering various cross-sectional configurations. Moreover, the subsequent stage of the research will involve assessing the level of crack resistance in the resulting materials by conducting tests to determine the stress intensity factor and comparing these findings with the corresponding characteristics of the base SiC ceramics.

## Figures and Tables

**Table 1:** Mechanical test data.

Material	Volume Fraction of Metal in Metal–Carbon Matrice, vol.%	Average Failure Load, N
SiC/C/Si (55/30/15 vol.%)	0	127.79
SiC/C/Si/Ti/TiSi_2_	13.3	134.73
SiC/C/Si/Nb/NbSi_2_	16.7	161.51
SiC/C/Si/Mo/MoSi_2_	16.7	178.94

## Data Availability

Data are contained within the article.
